# The utility of magnetic resonance angiography in children with nutcracker syndrome

**DOI:** 10.3906/sag-2101-14

**Published:** 2021-10-21

**Authors:** Dilara ATASOY, Ayşegül CANSU, Ayşe Füsun BEKİRÇAVUŞOĞLU, Elif BAHAT ÖZDOĞAN, Ali AHMETOĞLU

**Affiliations:** 1 Department of Radiology, Sivas Numune Hospital, Sivas Turkey; 2 Department of Radiology, Faculty of Medicine, Karadeniz Technical University, Trabzon Turkey; 3 Department of Radiology, Ministry of Health Bursa City Hospital, Bursa Turkey; 4 Division of Pediatric Nephrology, Department of Pediatrics, Faculty of Medicine, Karadeniz Technical University, Trabzon Turkey

**Keywords:** Nutcracker syndrome, nutcracker phenomenon, SMA angle, abdominal MRA

## Abstract

**Background/aim:**

The presented study aimed to evaluate the utility of magnetic resonance angiography (MRA) in the pediatric population with nutcracker syndrome.

**Materials and methods:**

Patients with suggestive clinical symptoms and laboratory findings and got the diagnosis of nutcracker syndrome with Doppler ultrasonography between January 2011–2019 were included in the study. In addition, children who had renal MRA due to hypertension were evaluated as the control group. MRA images of all patients were examined retrospectively by three radiologists at different levels of experience, and the superior mesenteric artery angle, aorta-mesenteric distance, left renal vein diameter both in the regions of aorta-mesenteric, and renal hilum were recorded.

**Results:**

Forty-five patients diagnosed with nutcracker syndrome were included in the study. The mean age of patients was 12 (4–16) and 30 (67%) were female. As the control group, 25 patients with hypertension who had MRA were included and they had a mean age of 12 (1–18) and 19 (76%) were male. The mean superior mesenteric artery angle was 26.5 ° (16–73 ± 12) in the patient group and 57.8 ° (25–139, ± 33) in the control group (p < 0.001); the mean aorta-mesenteric distance was 3.3 mm (1.7–6.5, ± 1.1) in the patient group and 8 mm (3.4–32, ± 5.9) in the control group (p < 0.001). MRA measurements of three radiologists were consistent with each other.

**Conclusion:**

MRA imaging can be applied as an alternative diagnostic method for Doppler ultrasonography and multidetector CT examinations by radiologists with different experience levels in pediatric patients with nutcracker syndrome.

## 1. Introduction

The nutcracker phenomenon is characterised by the compression of the left renal vein (LRV)–which can typically be between the superior mesenteric artery (SMA) and the abdominal aorta (the anterior nutcracker) or, less frequently, be between the aorta and the vertebral body (the posterior nutcracker). This phenomenon does not always present with clinical symptoms [1]. In patients with nutcracker syndrome (NCS), compression of the LRV results in left renal venous hypertension and related symptoms, such as flank pain, haematuria, pelvic congestion, left-sided varicocele, and orthostatic proteinuria [2].

In patients with clinical symptoms that are highly suspicious for NCS, a diagnosis of NCS can be validated with imaging techniques such as Doppler ultrasonography (DUS), multidetector CT (MDCT), magnetic resonance angiography (MRA), retrograde phlebography, and intravenous ultrasound. Phlebography and intravenous ultrasound are known as the gold standard methods of diagnosis; however, both are invasive methods and are rarely selected (only used if a diagnosis of NCS remains unclear with the other [noninvasive] techniques). The initial imaging technique that is mostly utilised is DUS. Following this, patients suspected of having NCS undergo cross-sectional angiographic imaging of the abdomen to demonstrate the anatomical relation of the LRV with the aorta and the SMA [3]. It seems that DUS has the disadvantages of patient and user dependency more prominently in pediatric patients than in adults. Also, a cross-sectional method such as MDCT is associated with ionisation radiation exposure, which makes it unsuitable for the pediatric patient group. Thus, we aimed to demonstrate the usefulness of MRA as a cross-sectional method in the diagnosis of NCS among pediatric patients.

## 2. Material and methods

### 2.1. Study population

Pediatric patients who had a diagnosis of NCS after clinical evaluation and DUS between January 2011 and January 2019 and had renal MRA were included as the patient group, while pediatric patients who had renal MRA to evaluate hypertension were categorized as the control group in this retrospective study. In the patient group, the children with persistent haematuria (microscopic/macroscopic) and/or proteinuria were clinically assessed for other common causes of these findings. If other differentials were excluded, then the patients were evaluated by DUS for possible NCS. The Doppler criterion for diagnosis in our radiology department was the LRV diameter and flow ratio (aortomesenteric/hilus) ≥ 4.9 [4]. Patients older than 18 years, without renal MRA or with conditions that may affect the SMA angle (para-aortic lymphadenopathies, intra-abdominal mass, intra-abdominal or retroperitoneal free fluid, and scoliosis) were excluded from the patient and control groups. In addition, patients with haematuria or proteinuria were excluded from the control group due to the possibility of undiagnosed NCS. The study was approved by the Local Research Ethics Committee, and the need for written informed consent was waived because retrospective data were used.

### 2.2. Image acquisition 

MRA imaging was performed using 1.5 T MR (Magnetom Symphony, Siemens Healthcare, Erlangen, Germany) and 3.0 T MR (Magnetom Skyra, Siemens Healthcare, Erlangen, Germany) machines. All MR sequences were acquired while the patients were in a supine position. The abdominal MRA imaging protocols of the study population for both 1.5 T MR and 3.0 T MR were contrast-enhanced 3D angiographic sequences. All image parameters were the same for the study and control group in both 1.5 and 3 T MR, except the contrast phase of the images. The images were acquired in the venous phase for the study group and the arterial phase for the control group. The imaging parameters used for each MR imaging sequence are demonstrated in Table 1.

**Table 1:** MRA imaging parameters.

Parameters	3T Siemens skyra	1.5 T Siemens symphony
Sequence	3D angio	3D angio
Image plane	Coronal	Coronal
TR (ms)	2.91	2.84
TE (ms)	1.02	1.14
Flip angle (°)	20	25
FOV (mm)	300	400
Slice thickness (mm)	1.1	1.4
Matrix	192X288	193X220
Number of slice	80	72
NEX	1	1
Total duration (s)	13	16
Contrast	0.1 mmol/kg	0.1 mmol/kg
Speed of injection (mL/s)	2.5	2.5

### 2.3. Image analysis

MR images were evaluated by three radiologists with different levels of experience (Radiologist 1: 3-year general radiologist, Radiologist 2: 5-year general radiologist, and Radiologist 3: 10-year abdominal radiologist). The radiologists were blinded to the groups. A picture archiving and communication system (Akgun PACS, Ankara, Turkey) was used for the analysis. The SMA angles were measured from the sagittal MRA sequences as the angle between the SMA origin, a point 1 cm along the posterior wall of the SMA and 1 cm along the anterior wall of the abdominal aorta [5]. The distance between the SMA and the abdominal aorta was measured on axial slices as the minimum distance between the anterior wall of the aorta and the posterior wall of the SMA at the level of the LRV. The caliper of the LRV at the aorta-mesenteric region (the narrowest point) and the left hilum region (the widest point) from the axial MRA sequence were measured, and the ratio between them (the LRV ratio) was calculated.

### 2.4. Statistical analysis

The Statistical Package for the Social Sciences for Windows (v: 23.0; IBM, New York, NY) was used for statistical analysis. Descriptive statistics of the evaluation results were given as mean value, standard deviation, minimum and maximum values for measured variables, and numbers (percentiles) for categorical variables. The one-sample Kolmogorov–Smirnov test was used to analyse the normal distribution of the data. Because the measured variables did not have a normal distribution, the Mann–Whitney U test was utilised for the comparison of the categorical variables of the patient and control groups (two independent groups). Bland–Altman plots were used to evaluate the agreement among the observers. The level of statistical significance was accepted as p < 0.05. The results of power analysis for the sample size of patients and control group with 95% confidence interval was 96%.

## 3. Results

The number of patients referred to the radiology department with suspicion of NCS was 70, but only 45 patients received a diagnosis of NCS after DUS. Of these patients, 64% clinically presented with proteinuria, 32% with haematuria and 4% with a combination of both. The participants in the patient and control groups that met the inclusion criteria were 45 and 25, respectively. The mean age at MRA examination was 12 in both groups. In the patient group, the female sex was dominant, whereas in the control group, there was male dominance (Table 2).

**Table 2:** The patient and control group in terms of demographics.

	Patient group	Control group
Number of subjects	45	25
Age	12(4–16)	12(1–18)
Sex	15 M (33%)30 F (67%)	19 M (76%)6 F (24%)

**Figure 1:**
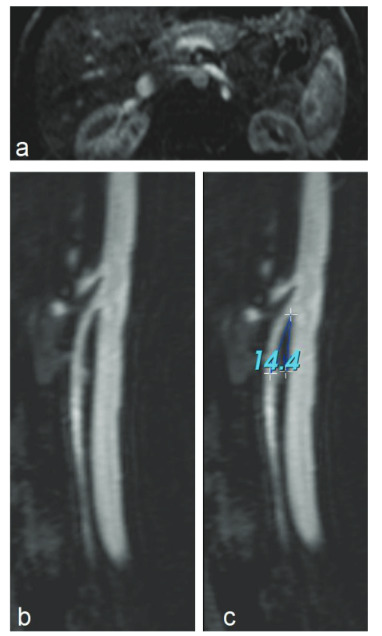


**Figure 2:**
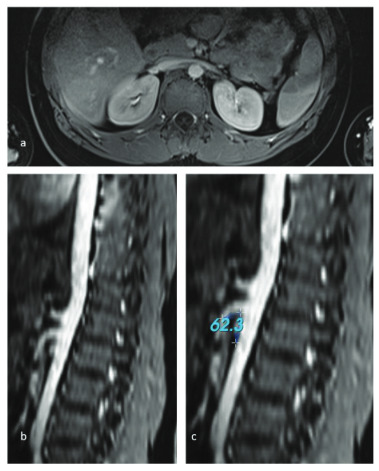


The mean aorta-mesenteric distance was 3.3 (1.7–6.5, ±1.1) mm in the patient group and 8 (3.4–32, ± 5.9) mm in the control group, and the distance was significantly narrower in patients with NCS (p < 0.001) (Figures 1a and 2a). Similarly, the SMA angle was 26.5 ° (16–73 ± 12) in the group with NCS, while it was 57.8 ° (25–139, ±33) in the control group (p < 0.001) (Figures 1b, 1c, 2b, and 2c). There was no statistically significant difference in renal vein diameter ratio between the two groups (Table 3).

**Table 3:** The patient and control group in terms of measurements.

Parameters	Patient group	Control group	P value
Aortamesenteric distance (mm)	3.3 (1.7–6.5, ±1.1)	8 (3.4–32, ±5.9)	p < 0.001
SMA angle (°)	26.5 (16–73, ±12)	57.8 (25–139, ±33)	p < 0.001
Renal vein ratio (hilus/aortomesenteric)	2.4 (1.42–7, ±1.3)	3.4 (1.1–3.4, ±0.9)	p > 0.05

**Figure 3:**
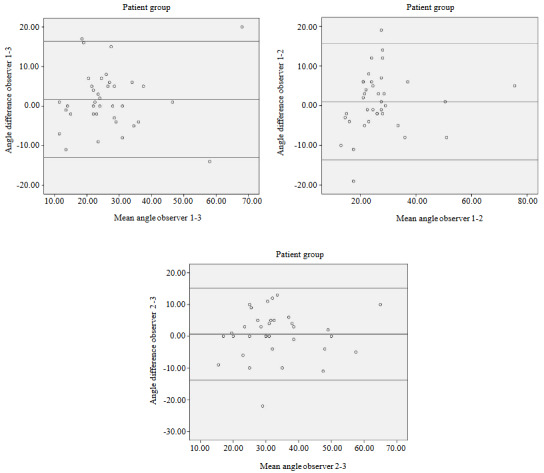


All the measurements were repeated by three observers, and interobserver variability was evaluated. Bland–Altman plots (Figure 3) revealed no significant differences between the three observers (Table 4).

**Table 4:** The difference between the radiologists in terms of measurements.

	SMA angle (°) difference mean ± STD	Aorta-mesenteric distance difference (mm) mean ± STD
Radiologist 1–2	0.95 ± 7.43 (–13.65, 15.55)	0.07 ± 1.02 (–1.92, 2.07)
Radiologist 2–3	0.73 ± 8.08 (–15.07, –16.56)	0.05 ± 0.87 (–1.65, 1.75)
Radiologist 1–3	1.67 ± 7.49 (–13.01, 16.35)	0.07 ± 0.74 (–1.38, 1.52)

## 4. Discussion

Although there is a lack of diagnostic consensus on NCS, imaging methods are commonly utilised for its diagnosis in patients with suspicious clinical presentations [1,4,6,7]. This study found that nutcracker-related measurements, including the SMA angle and the aorta-mesenteric distance, can be acquired from abdominal MRA to support the diagnosis of NCS in the pediatric population, similarly MDCT. In addition, this method can be used accurately by radiologists with different levels of experience.

Patients with NCS can belong to any age group – from the pediatric age group to the geriatric age group – however, the majority of the patients are young (second or third decade) and middle-aged adults [6]. In our study, we evaluated pediatric patients, in particular, owing to the need for a delicate diagnostic algorithm. Although the sex distribution is undetermined, it is estimated that the prevalence of NCS may be slightly higher in females [6]. Similarly, in this study, most of the participants in the patient group were females (67%).

NCS is mainly a clinical diagnosis, and the diagnosis should be made only in the presence of characteristic symptoms. Several imaging modalities can be used to confirm NCS, such as DUS, MDCT, MR-MRA, retrograde phlebography, and intravenous ultrasound [1,4]. Even though the sensitivity and specificity of DUS are variable (69–90 and 89–100, respectively) [6,8,9], it is considered the initial diagnostic method for NCS in patients with suspicious symptoms. It is noninvasive, has no radiation exposure and provides direct information about flow measurements [10,11]. However, insufficient patient cooperation during examinations and positional differences (supine, prone or upright position) may lead to variable results [12]. In our experience, the major challenge was a lack of cooperation during sonography with pediatric patients compared to adults. All DUS measurements were made in a supine position to minimise the inconsistency. Retrograde phlebography is known as the gold standard in the final diagnosis of NCS, but it is seldom chosen because it is invasive [3,13].

 MDCT angiography and conventional MR and MRA imaging provide more detailed information about the vascular anatomy of SMA region compared with sonography. However, one drawback of these imaging modalities is the inability to acquire direct information about flow dynamics. On the other hand, it is possible to acquire information about indirect haemodynamic consequences, such as prestenotic dilatation (hilar, periureteric, and pelvic varices) and dilated gonadal veins [3,4]. Additionally, MDCT angiography is associated with exposure to radiation and intravenous contrast material, which makes it questionable for pediatric patients. MR and MRA imaging can be the second choice after DUS in pediatric patients. Some conventional MR sequences, such as T1-VIBE, out-of-phase (opposed phase) T1, FSE T2WI, T2-TRUFI, and T2-HASTE sequences, may be useful for the diagnosis of NCS, with the benefit of not requiring contrast media exposure [14,15]. Although MRA angiography is not associated with radiation exposure, it still requires the usage of contrast material.

In our study, MRA revealed that the mean SMA angle in the patient population (26.5 °) was significantly lower than that of the control group (57.8 °). This is consistent with a study that found a significant difference in the SMA angle between children with and without NCS (17.8 ° vs. 28.7 °, respectively) on MDCT[16]. The largest cross-sectional (with MDCT) study of SMA angles in normal children reported the mean SMA angle as 45.6 °, which is lower than our control group. The study reported the mean distance between the aorta and the SMA as 8.6 mm, which is similar to our control group (8 mm) [5]. However, in this study, the mean distance between the aorta and the SMA was 3.3 mm in the patient group, which is significantly lower than the control group. Cho et al., also found a significant difference among children with and without NCS (4.3 mm vs. 6 mm, respectively) [16].

The mean renal vein ratio (hilus/aortomesenteric) was 2.4 mm in the patient group and 3.4 mm in the control group. Although it has been reported as the most specific measurement for NCS in MDCT in a previous study [6], we found no significant difference between the groups on MRA. However, we think that the results reflect the phase difference in MRA sequences between the patient group and the control group. Considering that the MRA images of our control group were obtained in the arterial phase, it was not possible to measure the vein ratio in most of our participants.

In this study, 3 radiologists with different levels of experience evaluated the images of the participants, and there was no significant difference in the measurements. We reckon that this is a benefit of MRA in the diagnosis of NCS in pediatric patients, especially when compared to DUS, which has a high user dependency. Although some studies have demonstrated value of the DUS in the diagnosis of NCS, in most of them, interobserver reproducibility was not calculated [8,11,12].

This study had some limitations. The main limitation was the usage of two different MR machines with different magnetic fields (1.5 T and 3 T) owing to the retrospective design of the study. Second, we defined the patients with hypertension as the control group because they were the only pediatric patient group with renal MRA in our department. Nevertheless, there were only a few cases in the literature with both NCS and hypertension, and it was mostly reported as a coincidence [17–20]. Additionally, there was male dominance in the control group, while most of the participants were females in the patient group. However, in a previous study, no significant sex difference was revealed in nutcracker-related measurements [5]. Finally, the phases of the MRA in the patient and control groups were different, which could be the reason for the statistically insignificant results in the measurement of the LRV diameter. In the patient group, the images were obtained in the venous phase, while they were obtained in the arterial phase in the control group. 

In conclusion, there is a lack of a diagnostic algorithm for NCS, which makes the diagnosis problematic. MR angiography provides a radiation-free alternative to CT angiography in children with NCS, with the ability to do the same diagnostic measurements of the SMA region. Moreover, it offers less user-dependent results compared to DUS. However, MRA still has the disadvantage of contrast media exposure; thus, there is a need for safer and objective diagnostic methods, and this could be evaluated in future research.

## Informed consent

The study was approved by our Local Research Ethics Committee and a need for written informed consent was waived as the retrospective data were used (the report number: 2019/19 of Karadeniz Technical University Ethics Comitte)
